# Supplementary material for: Under-screened Aboriginal and Torres Strait Islander women’s perspectives on cervical screening
Source: PLoS One. 2022 Aug 31;17(8):e0271658. doi: 10.1371/journal.pone.0271658 (PMC9432770; doi:10.1371/journal.pone.0271658)
Supplement: S1 File — (DOCX) [file pone.0271658.s001.docx]

# Yarning Guide

Please note the Yarning guide contains only the questions pertaining to the analysis reported in “Under-screened Aboriginal and Torres Strait Islander women’s perspectives on cervical screening” by Butler, Lee, Anderson, et al. 2022 *PLOS One.*

**Knowledge about screening**

- Have you heard much about these screening or pap tests?
- Many people say that they don’t know much about cervical screening tests or pap tests or what they are for. Can you tell me what you understand this screening is for?
- Where did you hear about these screening tests?
- What sorts of things does the clinic tell you about screening tests?
- How does the conversation with your doctor go? What kinds of things do they say?
- Have you heard other stories from friends or family about these tests? What sorts of things have you heard?

**Reasons for not screening**

- Many women don't get screened, either because they don't get around to it, or don't want to. Can you tell me a bit about why you haven't had a screen test?

**Views of screening**

- Do you think that screening is something that would benefit you? In what ways?
- How do you feel about having to see a doctor to be screened?

**Decision-making**

- Do you feel you need more information about screening before you could decide about whether to have a screening test?
- What type of information would help - info about the procedure, reasons for screening or both? Something else?
- Do you make the decision about having a screening test by yourself or do you like to talk about it with someone else?
- Do other peoples’ views on whether you should have a test help you decide whether to do it or not?
- If yes, in what ways? If no, why is that?

**Community views of screening**

- Do you think that women in your community talk about screening at all?
- Are there things that people in your community say about screening? Either good or bad?
- What do you think about these things?
- Do these views affect you wanting to have a screening test?

**Experience with clinic**

- Do you go to this clinic for all of your health needs?
- Do you feel you have a good relationship with the doctor and other staff at this clinic?
- Can you tell me about any reminders you might receive when it is time to have a screen?
- Are these reminders from the clinic useful?
- Have you had any letters from the cervical screening program? Do you think these are useful?
- Have you ever thought about making an appointment to talk about screening at the clinic?

**Suggestions that would make it easier for women to screen**

- Some people have suggested that there could be a person at the clinic to help women navigate the screening process, from start to finish. This person would help women with screening and with follow-up after screening, and answer any questions women might have. What do you think about this idea?
- Are there things that would change your mind or make it easier for you to get a screen test?
- Is there anything the clinic could do to make it easier for you to get a screen test?

**Conclusion**

- Do you have anything else you would like to say about screening today?
