# Supplementary material for: Under-screened Aboriginal and Torres Strait Islander women’s perspectives on cervical screening
Source: PLoS One. 2022 Aug 31;17(8):e0271658. doi: 10.1371/journal.pone.0271658 (PMC9432770; doi:10.1371/journal.pone.0271658)
Supplement: Attachment — Submitted filename: Review for PONE-D-22-05427.pdf [file pone.0271658.s002.pdf]

Title: Under-screened Aboriginal and Torres Strait Islander women's perspectives on cervical screening

Manuscript Number PONE-D-22-05427

The manuscript describes the results of a qualitative study that examined the views and experiences of Aboriginal and Torres Strait Islander women who were never screened, under-screened, or recently-screened after a long interval with regard to cervical screening.

The introduction provides a comprehensive background and clear rationale for the study. The study approach is appropriate, and participants, data collection, and analyses are clearly described. The results add to the literature regarding barriers to screening for Indigenous women. The finding that a lack of cervical screening is related not to a lack knowledge about screening or decreased access (which is often believed to be the case) but to the fact that for some women the harms outweigh the benefits is novel. Study strengths and limitations are appropriately described. The manuscript is well written.

Very small changes –

line 61, change data is to data are.

lines 77, 82, 415, 426, please note that a complete sentence is required before a colon.

Line 145, change was developed to were developed.

With respect to requirements for publication:

1. The study presents the results of original research. Yes.

2. Results reported have not been published elsewhere. Yes.

3. Experiments, statistics, and other analyses are performed to a high technical standard and are described in sufficient detail. Yes.

4. Conclusions are presented in an appropriate fashion and are supported by the data. Yes.

5. The article is presented in an intelligible fashion and is written in standard English. Yes.

6. The research meets all applicable standards for the ethics of experimentation and research integrity. Yes, ethical approvals are described in the manuscript.

7. The article adheres to appropriate reporting guidelines and community standards for data availability. Yes.
